# Chiral and Catalytic Effects of Site-Specific Molecular Adsorption

**DOI:** 10.1021/acs.jpclett.2c03575

**Published:** 2023-02-17

**Authors:** Bogdana Borca, Tomasz Michnowicz, Fernando Aguilar-Galindo, Rémi Pétuya, Marcel Pristl, Verena Schendel, Ivan Pentegov, Ulrike Kraft, Hagen Klauk, Peter Wahl, Andrés Arnau, Uta Schlickum

**Affiliations:** ∇ Max Planck Institute for Solid State Research, 70569 Stuttgart, Germany; ‡ National Institute of Materials Physics, Atomistilor 405A, 077125 Magurele, Ilfov, Romania; § Donostia International Physics Center, E-20018 Donostia - San Sebastián, Spain; ∥ Max Planck Institute for Polymer Research, Mainz 55128, Germany; ⊥ SUPA, School of Physics and Astronomy, University of St Andrews, North Haugh, St Andrews KY16 9SS, United Kingdom; # Departamento de Polímeros y Materiales Avanzados: Física, Química y Tecnología UPV/EHU and Material Physics Center (MPC), Centro Mixto CSIC-UPV/EHU, E-20018 Donostia - San Sebastián, Spain; 7 Institute of Applied Physics and Laboratory for Emerging Nanometrology, Technische Universität Braunschweig, 38104 Braunschweig, Germany

## Abstract

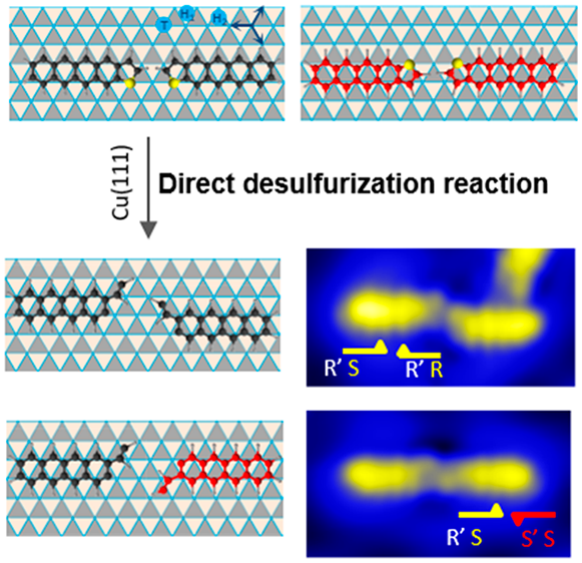

The changes of properties and preferential interactions based on subtle energetic differences are important characteristics of organic molecules, particularly for their functionalities in biological systems. Only slightly energetically favored interactions are important for the molecular adsorption and bonding to surfaces, which define their properties for further technological applications. Here, prochiral tetracenothiophene molecules are adsorbed on the Cu(111) surface. The chiral adsorption configurations are determined by Scanning Tunneling Microscopy studies and confirmed by first-principles calculations. Remarkably, the selection of the adsorption sites by chemically different moieties of the molecules is dictated by the arrangement of the atoms in the first and second surface layers. Furthermore, we have investigated the thermal effects on the direct desulfurization reaction that occurs under the catalytic activity of the Cu substrate. This reaction leads to a product that is covalently bound to the surface in chiral configurations.

How molecules adsorb on crystalline surfaces is a key element in designing and producing different molecular systems, as well as in controlling their functionalities. The main factors that define the molecular adsorption characteristics are the molecule–surface interaction and the energetics of dynamic processes on surfaces (such as diffusion, conformational changes, rotations, *etc.*) and the intermolecular interactions.^1^ These factors play a fundamental role in self-assembly and the bottom-up growth techniques for producing macromolecular systems^2−5^ and molecular structures with various geometries^2,6−14^ and chirality.^4,7,8,12,15−20^ These arrangements are of high interest for applications in molecular electronics,^6,9,10,18,19,21−25^ optoelectronics,^26−28^ adsorption-induced molecular magnetism,^29−31^ as well as molecular surface chemistry and catalysis.^2,5,26^

Furthermore, the adsorption properties associated with induced catalytic activity at metallic surfaces can be responsible for the formation of new molecular structures and materials.^32−34^ A particular example is the desulfurization reaction, required for the removal of sulfur atoms from crude oils, due to the harmful effects of sulfur compounds in the natural environment.^35−37^ In previous studies, we have investigated the electric-field-induced desulfurization reaction of tetracenothiophene (TCT) molecules adsorbed on a Cu(111) surface^38^ and its effect on the molecular conductance across the molecular derivative product TC-D.^39^ Here, we analyze the adsorption characteristics and the effect of the interaction of TCT and TC-D molecules with the surface and subsurface substrate atoms on the surface-induced chirality. In addition, quantitative details on the thermally induced desulfurization reaction of the thiophene group of the TCT are provided.

*Tetracenothiophene Adsorption on Cu(111)*. Intact TCT molecules (see molecular structure in Figure 1a) are deposited in a submonolayer regime onto the Cu(111) surface, maintaining the substrate temperature below 280 K. The molecules are adsorbed with their long axis along the high-symmetry crystallographic directions of the substrate (Figure 1a). In the topographic STM images, the molecules have an asymmetric “dumbbell-like” appearance, with the brighter side at the thiophene unit.^38,39^ The images were acquired at a temperature of about 6 K (Figure 1b).

**Figure 1:**
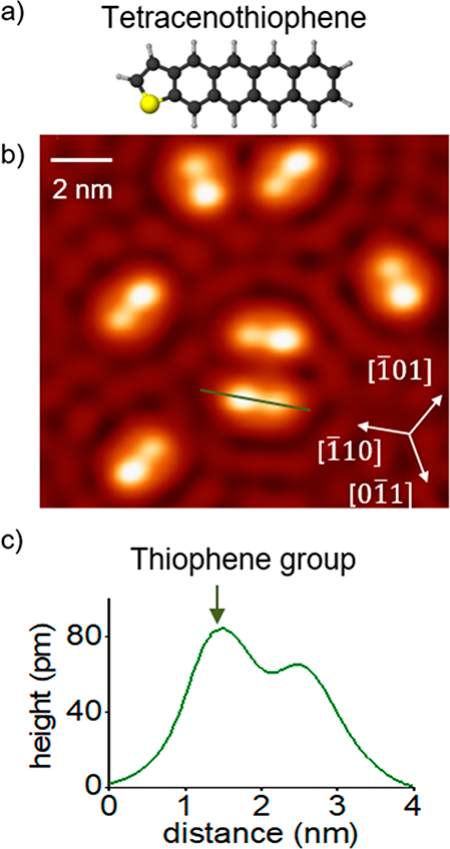
Tetracenothiophene (TCT) adsorption on Cu(111). (a) Molecular structure of TCT. (b) STM topography showing several TCT molecules adsorbed along the high-symmetry crystallographic directions of the Cu(111) surface. (c) Apparent height profile of a TCT molecule, with the highest side corresponding to the thiophene moiety.

The alignment of adsorbed TCT molecules with respect to the surface and the subsurface Cu atoms relies on the interplay between the positions of the two chemically different parts of the molecule, *i.e.*, the S-containing thiophene group and the acene group. This phenomenon is often encountered during the adsorption of organic molecules that have different functional groups.^8−11,17,18,29,40^ The aromatic rings of organic molecules adsorb mostly centered on the hollow sites of a (111) crystal surface.^1,6,7,9,11,15,21,22,38,41,42^ Moreover, the fine details of the adsorption geometry may also depend on the subsurface structure,^1,7,9,11,15,22,24,40−42^ if fcc-hollow and hcp-hollow sites are not equivalent sites for preferable adsorption. Indeed, benzene units preferentially adsorb at hcp-hollow sites,^1,22,41,42^ and thiophene rings on Cu(111) have a tendency to adsorb with the S atoms on top of a Cu surface atom.^21,25,38,43,44^ Additionally, the geometry and strength of the interactions between the other functional groups and the surface or the intermolecular interactions between them might also induce misalignments and adsorption on the fcc-hollow site.^7,11,21,40^

To obtain a quantitative understanding of the adsorption configuration of TCT molecules and the registry with Cu surface atoms on Cu(111), we have performed a theoretical study of the different adsorption sites using density functional theory calculations that include van der Waals interactions. In Figure 2a, ball-and-stick models of the optimized adsorption conformations of TCT/Cu(111) are shown. The most stable configuration follows the preferential adsorption tendencies, in which the S atom is placed on a Cu-atop position, while the acene group has the benzene units centered on the hcp-hollow sites. The difference in the relative adsorption energy compared to the adsorption on the hollow-fcc is 97.9 meV (Figure 2b). In consequence, molecules preferentially adsorb along the high-symmetry crystallographic [−1 1 0] axis of the surface.

**Figure 2:**
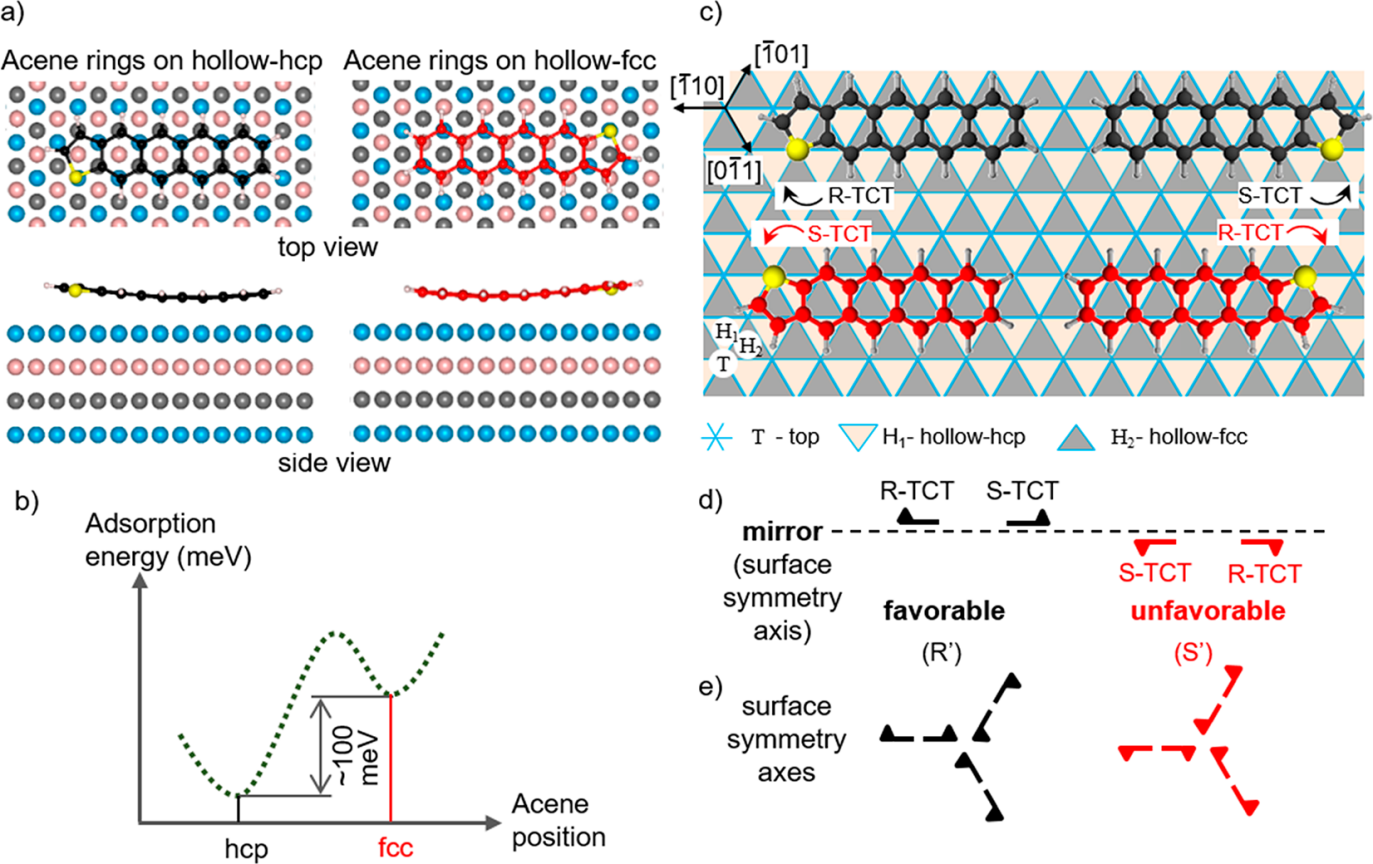
Preferential adsorption of TCT on Cu(111) and induced chirality. (a) Optimized adsorption geometry of TCT adsorption on the Cu(111) surface, revealing the favorable adsorption of the thiophene unit with the sulfur atom on top of a Cu atom and the acene rings on a hollow position, both the hollow-hcp and the hollow-fcc sites. (b) Relative adsorption energy difference between the two adsorption configurations, having the most stable arrangement on the hollow-hcp site. (c) Structural model of the TCT adsorption along one crystallographic direction of the Cu(111) surface resulting in a racemic mixture of R-TCT (clockwise) and S-TCT (anticlockwise) enantiomers with respect to the mirror planes perpendicular to the surface. (d) Schematic representation with half arrows of the R-TCT and S-TCT molecules. (e) Chiral geometry of TCT molecules adsorbed along the 3-fold symmetry axes of the surface with an R′ (clockwise) favorable and an S′ (anticlockwise) unfavorable configuration.

The prochiral TCT molecules, due to a surface-induced chirality, arise as “clockwise” R-TCT and “anticlockwise” S-TCT enantiomers (Figure 2c, d). Examining the molecules along one specific symmetry axis shows that the molecules on the hcp-hollow and fcc-hollow sites are mirror images with respect to this symmetry axis, since only then the requirement of the site-specific adsorption is fulfilled, with the S atom atop and acene rings on hollow positions (Figure 2c, d). In addition, considering the 3-fold symmetry of the surface and the selection rule of the two hollow positions for the molecular adsorption, the favorable hcp-hollow and unfavorable fcc-hollow sites can be assigned. This results in chiral geometries labeled R′ (clockwise) and S′ (anticlockwise) (Figure 2e). Besides the induced chirality, the adsorption sites are involved in the surface-activated TCT desulfurization, as presented in the following.

*Direct Desulfurization Reaction of Tetracenothiophene*. As previously mentioned,^39^ the adsorption of TCT on the Cu(111) surface at room temperature activates the cleavage of the S–C bonds from a portion of the adsorbed molecules and results in a desulfurized tetraceno derivative TC-D being strongly bound to the surface. In Figure 3a, the electronic states of the TCT and TC-D molecules close to the Fermi energy are mapped-out with a functionalized tip. For these measurements, a TCT molecule was attached from the surface to the tip apex, following a vertical manipulation procedure. As we have already shown,^38^ the desulfurization reaction leads to a reduction in the local density of states at the location of the pristine thiophene unit and the appearance of a darker feature at the former position of the S atom. The latter corresponds to the modification of the lowest unoccupied molecular orbital (LUMO) of the TCT and TC-D species, as the simulated STM image suggests^38^ (Figure 3b, c). Additionally, the sulfur atom is found to be repelled away from the molecule. An example of an aggregation that most likely corresponds to desulfurized S atoms is encircled in Figure 3a. In order to allow a strong bonding of the desulfurized derivative moiety, the reaction induces a slight misalignment of the TC-D acene rings with respect to the surface atoms (Figure 3b, c).

**Figure 3:**
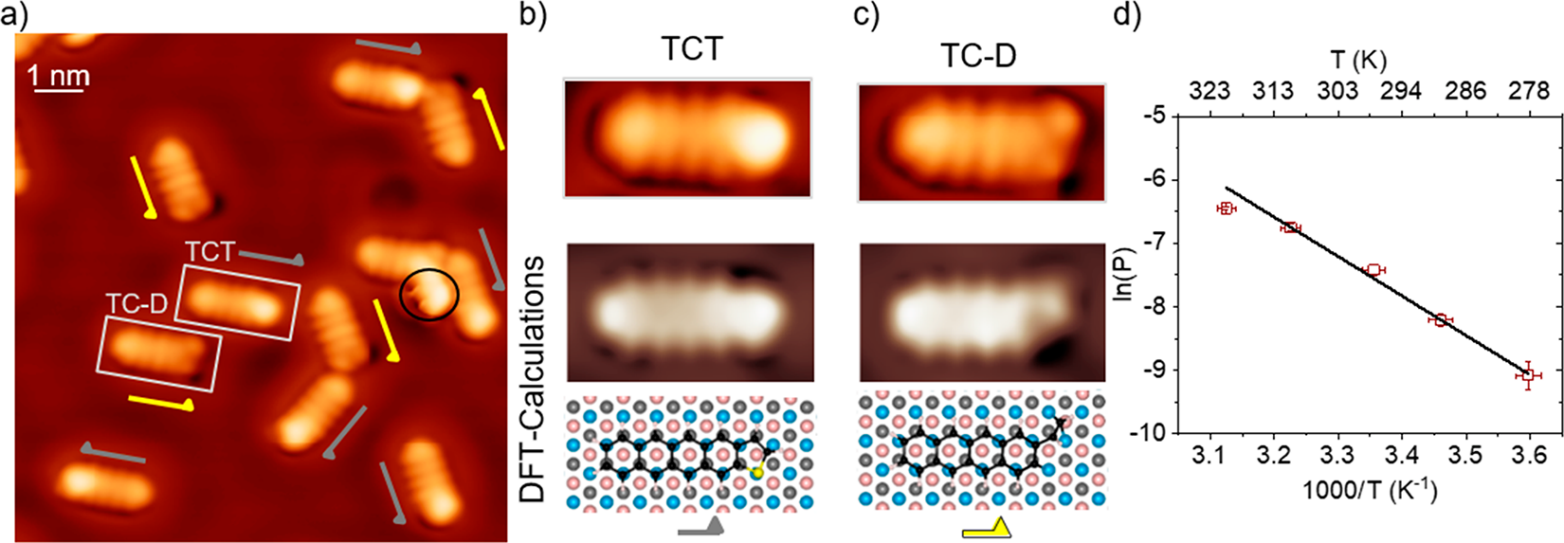
Thermally induced desulfurization reaction of TCT under the catalytic activity of Cu(111). (a) STM images acquired with a functionalized tip of the TCT and TC-D molecules following adsorption at a temperature of about 300 K. Half arrows mark each molecular isomer. (b, c) Zoom-in images (marked with rectangles in panel (a) together with the STM simulations in the Tersoff–Hamann approximation of the molecular aspect^38^ and of the calculated molecular structure of the TCT and TC-D molecules. (d) Arrhenius plot of the TCT desulfurization reaction.

Concerning the effects of temperature on the catalytically assisted desulfurization reaction of the thiophene group of the TCT molecules, the analysis of the reaction rate (*P*) as a function of the Cu substrate temperature (*T*) during the deposition shows a dependence that follows the Arrhenius law (Figure 3d). This is described by the equation: *P*, where *E*_a_ is the activation energy, *k*_B_ is the Boltzmann constant, and *A* is the pre-exponential factor. The activation energy determined experimentally from the Arrhenius plot is 0.50 ± 0.02 eV, a value close to the one obtained theoretically of about 0.66 eV.^38^ The extracted value of *A* is (6.22 ± 7.65) × 10^5^ s^–1^. The reaction is exothermic.^38^ The released energy contributes to the expulsion of the S atom from the molecule^38^ and may influence the reaction of the molecules in the close surrounding. If the coverage of TCT was increased, an increase of the reaction probability from 30–40% to 55–65% was observed (for details, see Supporting Information). In this case the molecular coverage was increased from approximately 10% to 50% on the Cu(111) surface kept at a temperature of about 300 K during adsorption.

A careful analysis of the orientation of the TC-D molecules at high coverage reveals that some molecules are located on the less convenient surface adsorption sites due to certain geometrical constraints. The high-resolution STM images in Figure 4 were acquired with a functionalized tip. Classifying the TC-D enantiomers leads to a majority of molecules (approximately 96%) arranged in the favorable configuration with the ethylene end group positioned on one side of the symmetry axes of the surface (R′ conformations of the R-TC-D and S-TC-D enantiomers). This is based on the selection rule associated with the preferential adsorption position at the hollow sites, as also confirmed by density functional theory calculations. However, if two molecules are extremely close to each other, one of the molecules adopts the less favorable arrangement with respect to the subsurface atoms, *i.e.*, the S′ conformation (Figure 4a, b, red marks). From the structural analysis of the data we assume that this arrangement is mediated by both molecule–surface and intermolecular interactions. The difference in the adsorption energy between the favorable hcp registry and the fcp registry of the acene rings of the molecules on the Cu(111) is relatively low. Thus, already weak intermolecular interactions can dominate over molecule-surface interaction and lead to the rearrangement of the adsorption geometry to the less favorable position.

**Figure 4:**
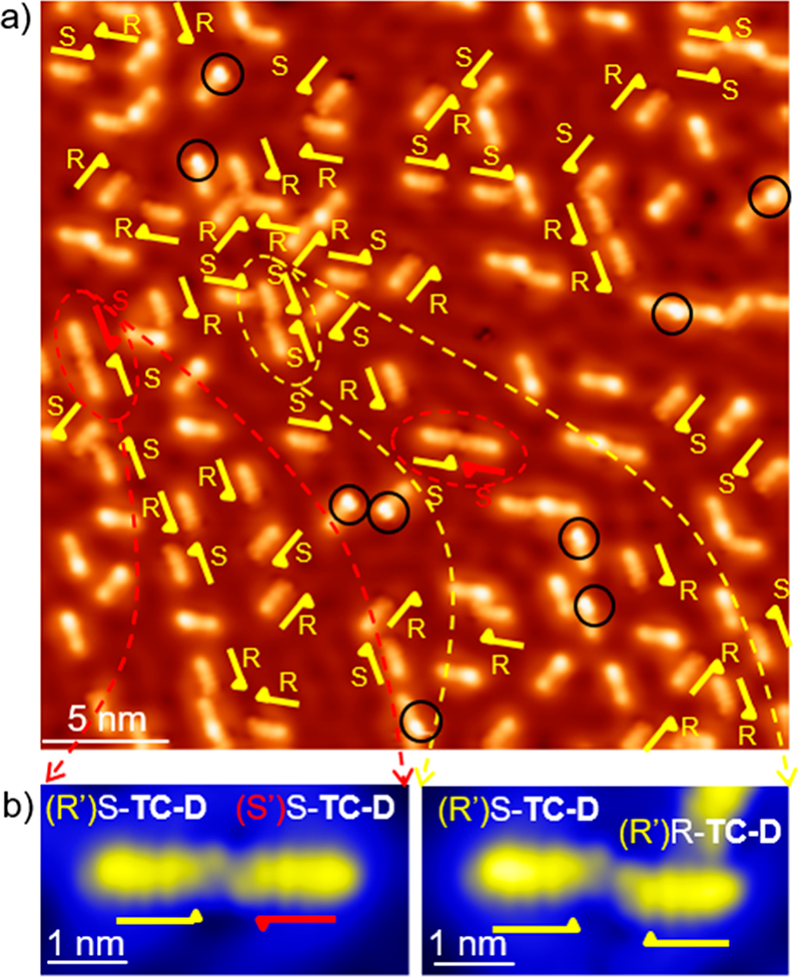
STM images of the racemic mixture of TCT and TC-D molecules. (a) Topographic overview image acquired with a functionalized tip. Yellow half arrows are used to mark the TC-D enantiomers. (b) Zoom-in images of TC-D dimers showing different occurrences, with an unfavorable S′ configuration (red marks) of a TC-D molecule in the left panel.

In summary, we have investigated the adsorption characteristics of TCT molecules and of molecules of the desulfurized derivative TC-D on Cu(111). Our studies demonstrate the preferential adsorption conformation being along the high-symmetry axes of the surface with the thiophene unit atop a Cu atom and the acene rings on the hcp-hollow sites. This is confirmed by density functional theory calculations. The preferred adsorption geometry induces a molecular chirality of right-handed and left-handed enantiomers of a prochiral building block and of the TC-D product. Additionally, as the molecular coverage is increased, certain constraints and competing interactions give rise to the formation of different aggregates with both types of enantiomers. Under these conditions, dimers and, occasionally, trimers with different orientations are formed as well. These findings open the possibility to grow superstructures with specific functionalities and chiral properties tunable by the marginal energetic changes in the adsorption conformation.

## Experimental and Computational Methods

*Experimental Procedures*. Tetraceno[2,3-*b*]thiophene (TCT) molecules were synthesized by following the processes described in detail in refs (39 and 45). The Cu(111) substrate was cleaned before each deposition by standard procedures of Ar^+^ ion etching and subsequent annealing to a temperature of 800 K. TCT was deposited under ultrahigh vacuum conditions by thermal sublimation onto the Cu(111) surface held at a constant temperature between 275 and 325 K during the deposition. In order to study the thermal effects on the desulfurization reaction, the deposition of an approximately identical amount of molecules corresponding to a surface coverage of about 10% was realized with the same rate and deposition time (10 min) onto the substrate held at the defined temperature. After each deposition, the sample was immediately transferred *in situ,* in a time span of a few minutes, into the STM operating at a cryogenic temperature of about 6 K. The STM measurements were carried out in constant-current mode by applying the bias voltages to the sample. For the determination of the reaction probability as a function of the substrate temperature during the deposition, a minimum of two separate depositions were carried out for each defined substrate temperature. Several STM images were acquired for each sample on different macroscopic regions containing more than 600 molecules. STM images with submolecular resolution were obtained with a functionalized tip having a TCT molecule attached by vertical manipulation to the tip apex. This process consists of first imaging the TCT molecule to be picked up. Then, the STM tip is placed on top of the molecule, the feedback loop is switched off, and the tip is approached toward the molecule. At a distance where attractive forces are dominant, the molecule attaches to and remains at the tip apex when the tip is lifted from the surface again.

The WSxM software^46^ was employed for the analysis of the STM images.

*Computational Methods*. Calculations were performed in the frame of density functional theory (DFT), under the generalized gradient approximation (GGA) with the vdW-DF-cx functional.^47^ This allows us to include van der Waals forces, with the Vienna ab initio simulation package (VASP).^48^

The electron density was expanded in a plane-wave basis with a cutoff energy of 500 eV, and the interaction between electrons and nuclei was described with the projector augmented wave (PAW) pseudopotentials from the VASP database. Reciprocal space was sampled using a 3 × 3 × 1 k-mesh, following the Monkhorst–Pack scheme.

To simulate the adsorption of the molecules on the Cu(111) surface, we used a supercell that consists of a 4-layer slab of 8 × 8 Cu atoms. A vacuum gap with a separation of ∼15 Å was used to avoid spurious interaction between the molecule and the closest replica.

We used a convergence criterion of 10^–5^ eV for the electron density during optimizations. We consider a structure as converged when all the Hellman–Feyman forces are smaller than 0.01 eV/Å, for the degrees of freedom that we allow to relax (*xyz* coordinates of all the atoms in the molecule and *z* of the outermost metal layer).

The VESTA software^49^ was employed for the ball-and-stick representations.

## Supporting Information Available

Experimental results on the desulfurization reaction of TCT molecules by adsorption at a temperature of about 300 K on Cu(111) (PDF)
