# Supplementary material for: Chiral and Catalytic Effects of Site-Specific Molecular Adsorption
Source: J Phys Chem Lett. 2023 Feb 17;14(8):2072–7. doi: 10.1021/acs.jpclett.2c03575 (PMC9986952; doi:10.1021/acs.jpclett.2c03575)
Supplement: Supplementary file 1 — jz2c03575_si_001.pdf [file jz2c03575_si_001.pdf]

Supporting information

Chiral and Catalytic Effects of Site-Specific Molecular Adsorption

Bogdana Borca,[1,2] Tomasz Michnowicz,[1] Fernando Aguilar-Galindo,[3] Rémi Pétuya,[3,#] Marcel Pristl,[1] Verena Schendel,[1] Ivan Pentegov,[1] Ulrike Kraft,[1,4] Hagen Klauk,[1] Peter Wahl,[1,5] Andrés Arnau[3,6] and Uta Schlickum[1,7*]*

[1]Max Planck Institute for Solid State Research, 70569 Stuttgart, Germany

[2]National Institute of Materials Physics, Atomistilor 405A, 077125 Magurele, Ilfov, Romania

[3]Donostia International Physics Center, E-20018 Donostia - San Sebastián, Spain

[4]Max Planck Institute for Polymer Research, Mainz 55128, Germany

[5]SUPA, School of Physics and Astronomy, University of St Andrews, North Haugh, St Andrews, KY16 9SS, United Kingdom

[6]Departamento de Física de Materiales UPV/EHU and Material Physics Center (MPC), Centro Mixto CSIC-UPV/EHU, E-20018 Donostia - San Sebastián, Spain

[7]Institute of Applied Physics and Laboratory for Emerging Nanometrology, Technische Universität Braunschweig, 38104 Braunschweig, Germany

Present address: [#]Nextmol (Bytelab Solutions SL), Barcelona 08018, Spain

* bogdana.borca@infim.ro, u.schlickum@tu-bs.de

Direct desulfurization reaction of tetracenothiophene on Cu(111) – coverage effect

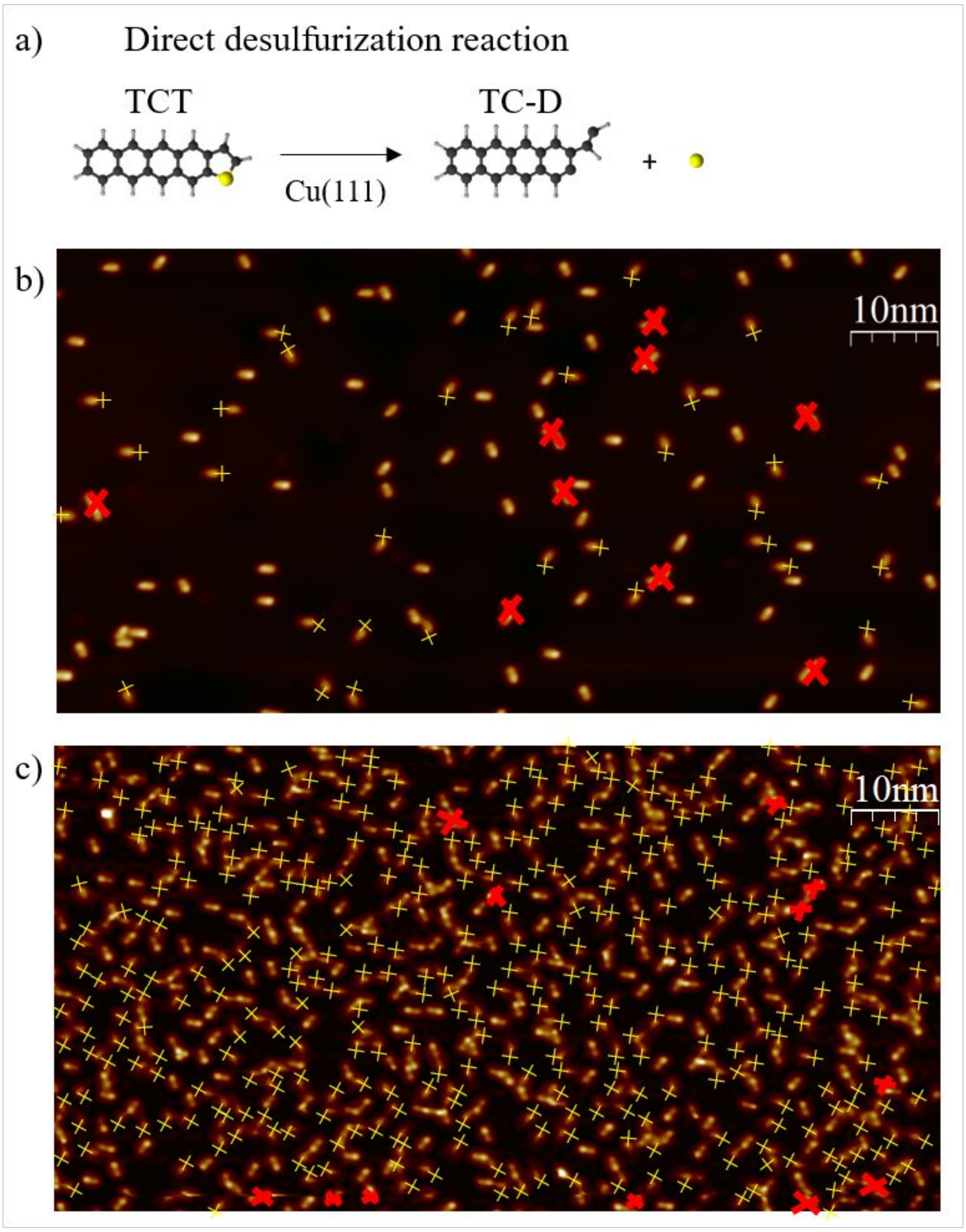

Figure S1. Dependence of the probability of the direct desulfurization reaction of tetracenothiophene (TCT) on the molecular surface coverage for adsorption at a temperature of approximately 300 K. a) Schematic representation of the molecules involved in the reaction. b) STM image for a coverage of about 10%, which results in about 33% tetraceno-derivative (TC-D). c) STM image for a coverage above 50%, resulting in about 59% TC-D. TC-D

molecules are marked with a yellow cross. Molecular defects excluded from the statistics are marked with a large red cross.

The catalytic properties of a Cu(111) surface favor the activation of a direct desulfurization reaction of the thiophene moiety in tetracenothiophene (TCT) molecules (Figure S1a) adsorbed on the Cu(111) surface, even at temperatures below 300 K. The desulfurization reaction produces a tetraceno-derivative TC-D (Figure S1a) bonded to the surface, which is characterized in scanning tunneling microscopy (STM) images by a darker feature corresponding to the reduction of the local density of states at the former position of the S atom in the intact thiophene unit of a TCT molecule (Figure S1b,c).

The reaction is exothermic, in agreement with the studies of the desulfurization reaction induced in TCT by the action of an electric field.[1] The released energy contributes to the repulsion of the resulting S atoms away from the TC-D molecules, but also to the enhancement of the desulfurization rate. Thus, by increasing the molecular coverage of TCT molecules adsorbed on Cu(111) at room temperature (300 K) from about 10% to about 50%, the desulfurization reaction probability increases from about 30-40% to about 55-65% (Figure S1b-c).

REFERENCES

(1) Borca, B.; Michnowicz, T.; Pétuya, R.; Pristl, M.; Schendel, V.; Pentegov, I.; Kraft, U.; Klauk, H.; Wahl, P.; Gutzler, R.; Arnau, A.; Schlickum, U.; Kern, K. Electric-Field-Driven Direct Desulfurization. *ACS Nano,* **2017**, *11*, 4703-4709. https://doi.org/10.1021/acsnano.7b00612
